# Supplementary material for: Typhi Mykrobe: fast and accurate lineage identification and antimicrobial resistance genotyping directly from sequence reads for the typhoid fever agent Salmonella Typhi
Source: bioRxiv. 2025 Jul 31:2024.09.30.613582. Preprint. [Version 2] doi: 10.1101/2024.09.30.613582 (PMC12324182; doi:10.1101/2024.09.30.613582)
Supplement: Supplement 1 [file media-1.pdf]

Supplementary materials

Ingle et al.

Typhi Mykrobe: fast and accurate lineage identification and antimicrobial resistance genotyping directly from sequence reads for the typhoid fever agent *Salmonella* Typhi

Supplementary tables, and code to generate tables and figures, is in the Typhoid Genomics Consortium Typhi Mykrobe github:
https://github.com/typhoidgenomics/TyphoidGenomicsConsortiumMykrobe

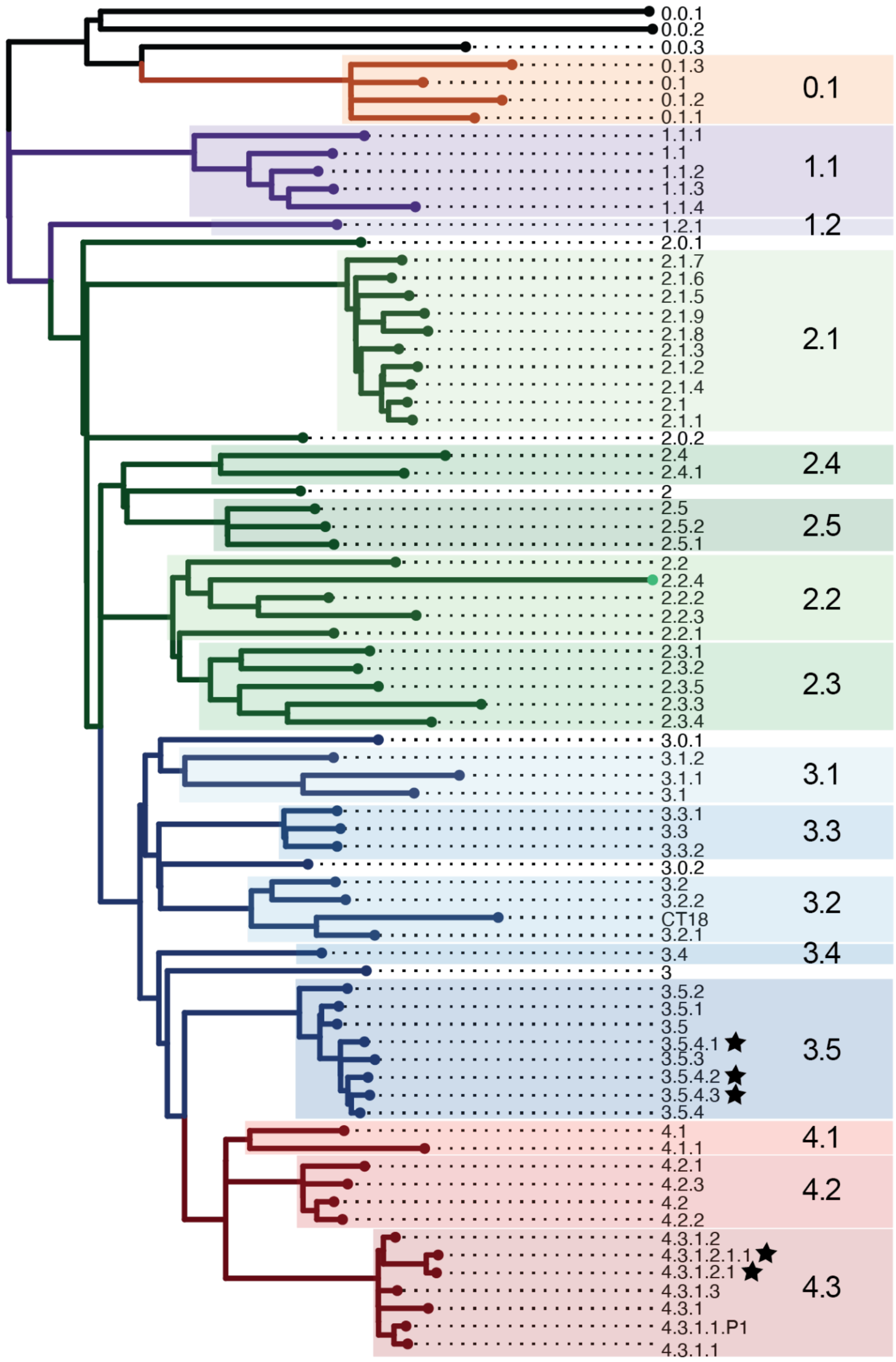

Supplementary Figure 1: Overview of the GenoTyphi scheme
Phylogenetic tree backbone showing the relationships between the lineages, clades and subclades. Tree tips represent unique genotypes as labeled, and background shading highlights clades (labeled in larger font). The black stars indicate genotypes added to the scheme in the 2022 Technical Report (https://zenodo.org/doi/10.5281/zenodo.7407984)

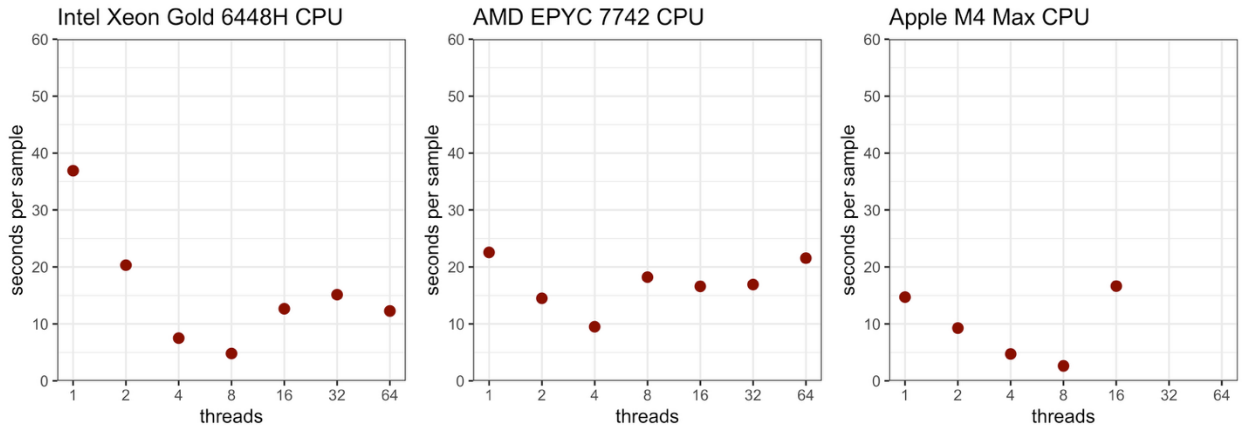

Supplementary Figure 2: Average run-time of Typhi Mykrobe of 100 Typhi genomes
The average run time for 100 Typhi genomes on three different computers. The number of threads used is shown on the x-axis. The average time (seconds per sample) to run on each genome is shown on the y axis.

Supplementary Table 1. Tabulated Typhi Mykrobe output table for all genomes included in validation analyses (available in github).

Supplementary Table 2: Genome data used for validation (available in github).

Supplementary Table 3. Details of AMR genotype calls comparison (available in github).

Supplementary Table 4. Genome data for all isolates with publicly available antimicrobial susceptibility testing (AST) (available in github).

Supplementary Table 5. Comparison and error rates for AMR genotype and phenotype data (available in github).

Supplementary Table 6. Details of validation of typing from nanopore reads (available in github).

Supplementary Table 7. Validation of AMR genotyping from ONT reads (vs Illumina)

Drug	Illumina	ONT (vs Illumina)	N	Agreement
Ampicillin	*bla*TEM-1	agree	35	100%
	no marker	agree	57	
Azithromycin	no marker	agree	92	100%
Ceftriaxone	*bla*CTX-M-15	agree	5	100%
	*bla*SHV-12	agree	1	
	no marker	agree	86	
Chloramphenicol	*catA1*	agree	25	98.99%
		*no marker	1	
	no marker	agree	66	
Ciprofloxacin	1 QRDR	agree	34	91.30%
		*different QRDR	1	
		*no marker	3	
	2 QRDR	agree	2	
	3 QRDR	agree	3	
	1 QRDR + *qnr*	agree	5	
		*1 QRDR	1	
	no marker	agree	40	
		*1 QRDR	3	
Sulfonamides	*sul1*	agree	5	100%
	sul2	agree	9	
	sul1;sul2	agree	24	
	no marker	agree	54	
Trimethoprim	*dfrA7*	agree	26	100%
	dfrA14	agree	4	
	dfrA15	agree	2	
	no marker	agree	60	
Tetracycline	*tetA(A)*	agree	7	100%
	tetA(B)	agree	2	
	no marker	agree	83	

Supplementary Table 8. Validation of plasmid marker detection from ONT reads (vs Illumina)

Rep marker	Illumina	ONT (vs Illumina)	N	Agreement
IncFIAHI1	present	agree	1	100%
	absent	agree	91	
IncHI1A	present	agree	1	98.91%
	present	*absent	1	
	absent	agree	90	
IncHI1BR27	present	agree	2	100%
	absent	agree	90	
IncHI1_ST6	present	agree	2	100%
	absent	agree	90	
IncHI2A	absent	agree	92	100%
IncY	present	agree	7	100%
	absent	agree	85	
IncX3	present	agree	1	100%
	absent	agree	91	
IncI1	absent	agree	92	100%
IncL_M	absent	agree	92	100%
IncFIB_pHCM2	present	agree	12	96.74%
	present	*absent	3	
	absent	agree	77	
IncFIB_K	present	agree	2	100%
	absent	agree	90	
IncN	present	agree	3	100%
	absent	agree	89	
z66	absent	agree	92	100%
**Total**	present	agree	31	**99.67%**
	present	*absent	4	
	absent	agree	1161	
